# Cardiac disorders associated with compound long-acting bronchodilators for inhalation: a pharmacovigilance analysis of the FDA adverse event reporting system database

**DOI:** 10.3389/fcvm.2026.1715192

**Published:** 2026-03-09

**Authors:** Ying Lan, Die Hu, Shijing Huang, Min Xu, Qin He

**Affiliations:** Department of Pharmacy, The Affiliated Hospital of Southwest Jiaotong University, The Third People's Hospital of Chengdu, Chengdu, Sichuan, China

**Keywords:** adverse cardiac events, FAERS, LABA, LAMA, long-acting anticholinergic drugs, long-acting beta 2 receptor agonists, pharmacovigilance analysis

## Abstract

**Background:**

This study aimed to utilize the FDA Adverse Event Reporting System (FAERS) database to systematically investigate the relationship between compound inhaled long-acting bronchodilators for inhalation (ILABs) and cardiovascular adverse events.

**Methods:**

We conducted a pharmacovigilance analysis using cardiac adverse event (CAE) reports submitted to FAERS between January 2014 and September 30, 2024. We compared the cardiac toxicity signal intensity of various compound ILABs using the proportional imbalance measurement method. Additionally, we performed subgroup analyses considering factors such as composition, onset time, mortality outcome case information, and concomitant adverse events.

**Results:**

Our study comprised 3,120 reports on the use of compound ILABs, involving 653 CAEs (454 reports). The most prevalent CAEs observed were atrial fibrillation (14.93%), myocardial infarction (14.37%), cardiac failure congestive (10.96%), cardiac disorder (8.70%), and cardiogenic shock (8.13%). Approximately 20% of CAEs occurred on day 0, and roughly 50% occurred within 90 days. The proportion of CAE reports was similar for triple inhalation formulations and double bronchodilators. But the number of adverse events such as congestive cardiac failure, coronary artery disease, and ventricular extrasystoles associated with triple inhaled formulations was significantly higher than that with dual bronchodilators, with no such reports for the latter. 84.58% of CAE cases were linked to other adverse events.

**Conclusion:**

Compound ILABs had significant CAE risk signals, especially those of atrial fibrillation, myocardial infarction and heart failure, and the risk signal was stronger in the early stage of drug treatment. Except for a few individual adverse events, the overall CAE risk signal associated with triple inhaled agents (mainly Fluticasone/umeclidinium/vilanterol) was comparable to that of dual bronchodilators.

## 1 Introduction

Chronic obstructive pulmonary disease (COPD) is a widespread respiratory condition characterized by high prevalence, mortality, and a significant disease burden (http://www.who.int/) (1–3). By 2050, projections indicate that the global number of COPD patients will reach approximately 600 million, with a notable surge among women and in low-income nations. Currently, COPD claims the lives of around 3 million people annually, a figure that is anticipated to rise to 5.4 million by 2060 (https://goldcopd.org/2025-gold-report/). Inhaled long-acting bronchodilators (ILABs), including single-agent formulations of long-acting *β*2 agonists (LABA) or long-acting muscarinic antagonists (LAMA), as well as their combination formulations, constitute the primary pharmacological treatment for stable COPD. Given the requirement for long-term or lifelong therapy, the safety profile of ILABs, particularly their potential cardiac toxicity, has garnered considerable attention. Multiple clinical studies had also confirmed that monotherapy with LABA or LAMA increases the risk of cardiovascular events (e.g., cardiac arrhythmias, acute coronary syndrome, heart failure, ischaemic stroke) (4–7), and the risks of such cardiovascular events induced by LABA and LAMA were comparable (8). Consequently, the cardiac toxicity associated with compound preparations containing both LABA and LAMA warrants heightened awareness.

The FAERS database comprises self-reported adverse events from numerous countries, updated quarterly. As of the third quarter of 2024, it had offered more than 20 million adverse event reports, enabling the detection of rare adverse reactions and the exploration of novel adverse reaction signals (9, 10). By employing data mining techniques, we can systematically analyze the FAERS database, thereby addressing limitations inherent in traditional clinical trials, such as small sample sizes, brief observation periods, and the exclusion of patients with heart disease. This study aims to utilize these techniques to comprehensively investigate the cardiac toxicity signals associated with compound ILABs within the FAERS database. Our objective is to elucidate the characteristics and risk factors of these drugs' cardiac toxicity, ultimately providing a scientific foundation for clinical decision-making, drug risk management, and further mechanistic investigations.

## 2 Materials and methods

### 2.1 Data sources and processing

We conducted a pharmacovigilance study to investigate cardiac adverse events associated with compound ILABs, utilizing data from the FAERS database. We determined the search start time based on the approval dates of each drug by the US Food and Drug Administration, as detailed in Table 1, with a search cutoff date of September 30, 2024 (https://www.accessdata.fda.gov/scripts/cder/daf/index.cfm).

**Table 1:** The time of FDA approval and the start time of FAERS database search for each drug.

Generic name	Brand name	FDA approval time	Search start time
Formoterol/aclidinium	DUAKLIR PRESSAIR	03/29/2019	Q2 2019
Formoterol/glycopyrrolate	BEVESPI AEROSPHERE	04/25/2016	Q2 2016
Indacaterol/glycopyrrolate	UTIBRON	10/29/2015	Q4 2015
Olodaterol/tiotropium	STIOLTO RESPIMAT	05/21/2015	Q3 2015
Vilanterol/umeclidinium	ANORO ELLIPTA	12/18/2013	Q1 2014
Budesonide/glycopyrrolate/formoterol	BREZTRI AEROSPHERE	07/23/2020	Q3 2020
Fluticasone/umeclidinium/vilanterol	TRELEGY ELLIPTA	09/18/2017	Q4 2017

For data management, we used Navicat Premium 15.0.12 to connect to MySQL, facilitating filtering, extraction, merging, deduplication, exporting, and other data manipulation operations. Given that some adverse event reports have been submitted to the FDA multiple times with updated information, it was necessary to reprocess the database data. Following the FDA's recommended approach, we eliminated duplicate reports to ensure data cleanliness and analytical accuracy, keeping only the most recent version of each report.

The FAERS database comprises seven distinct data tables (11). These include: (1) DEMO, containing patient demographics and management information; (2) DRUG, detailing major and minor suspicious drugs, concomitant drugs, and interacting drugs; (3) REAC, documenting adverse events; (4) OUTC, recording patient outcomes; (5) RPSR, indicating report sources; (6) THER, specifying drug treatment start and end dates; and (7) INDI, listing indications for use or diagnosis. All these tables are linked by a unique “PRIMARYID” field. From the DEMO data, we extracted the PRIMARYID, age, weight, gender, reporter occupation type, country of event occurrence, and reporting year for each case. Adverse drug events linked to each PRIMARYID were sourced from the REAC data, while the ultimate outcomes after these events were retrieved from the OUTC data.

Drawing from FDA drug registration records, the present study focused on double bronchodilators, specifically including combinations such as Formoterol/aclidinium (FA), Formoterol/glycopyrrolate (FG), Indacaterol/glycopyrrolate (IG), Olodaterol/tiotropium (OT), and Vilanterol/umeclidinium (VU). Additionally, the study encompassed fixed-dose triple inhalation preparations, namely budesonide/glycopyrrolate/formoterol (BGF) and Fluticasone/umeclidinium/vilanterol (FUV). To screen for adverse events caused by these drugs, we utilized the “Drug” data table. Specifically, we filtered the data based on the “DRUGNAME” or “prod_ai” fields containing the generic and product names listed in Table 1, ensuring that the `role_cod` field was set to `PS` (primary check drug). To retrieve data on adverse events related to formoterol/aclidinium bromide, a fuzzy search was conducted in the `drugnamè or `prod_aì fields for entries including `Formoterol/aclidinium` and `DUAKLIR PRESSAIR`. Adverse events and medication errors in the report were coded using preferred term (PT) codes from the ICH International Dictionary of Medical Terminology (MedDRA, version 27.0, https://www.meddra.org/how-to-use/support-documentation/english) and mapped to the corresponding System Organ Class (SOC).

### 2.2 Statistical analysis

In pharmacovigilance research, disproportionality analysis serves to evaluate potential associations between specific adverse events and particular drugs. By comparing data from the FAERS database, a notably elevated frequency of adverse events linked to the drug under investigation, reaching a predefined critical threshold (a < 2), suggests a significant adverse reaction signal (12). Our study adopts the frequency method commonly used in the proportional imbalance measurement method, such as the reported odds ratio (ROR) method, the proportional reported odds ratio (PRR) method, the medicines and healthcare products regulatory agency comprehensive standard method (MHRA method for short), and the Bayesian credible propagation neural network method (BCPNN) as the main method of ADEs signal mining (13–15). We used the four-grid table to calculate ROR, PRR, *χ*2 (chisquare), the 95% confidence interval (CI) and other statistical indicators (Supplementary Table S1). Specifically, “a” represents the count of target ADEs linked to the target drug, “b” denotes non-target ADEs for the same drug, “c” refers to target ADEs attributed to non-target drugs in the FAERS database, and “d” signifies non-target ADEs caused by non-target drugs. The threshold of CAEs signal in this study was set to meet the threshold criteria of PRR, ROR, MHRA and BCPNN algorithm at the same time, which was a > 2, the lower limit of 95% confidence interval of ROR and PRR > 1, PRR ≥ 2 and Chi-square (*χ*^2^) ≥ 4. We also compared the difference of CAEs signal intensity of the four algorithms. See Supplementary Table S1 for the calculation formula of each algorithm and the signal intensity classification standard.

## 3 Results

### 3.1 Descriptive analysis

**Figure 1:**
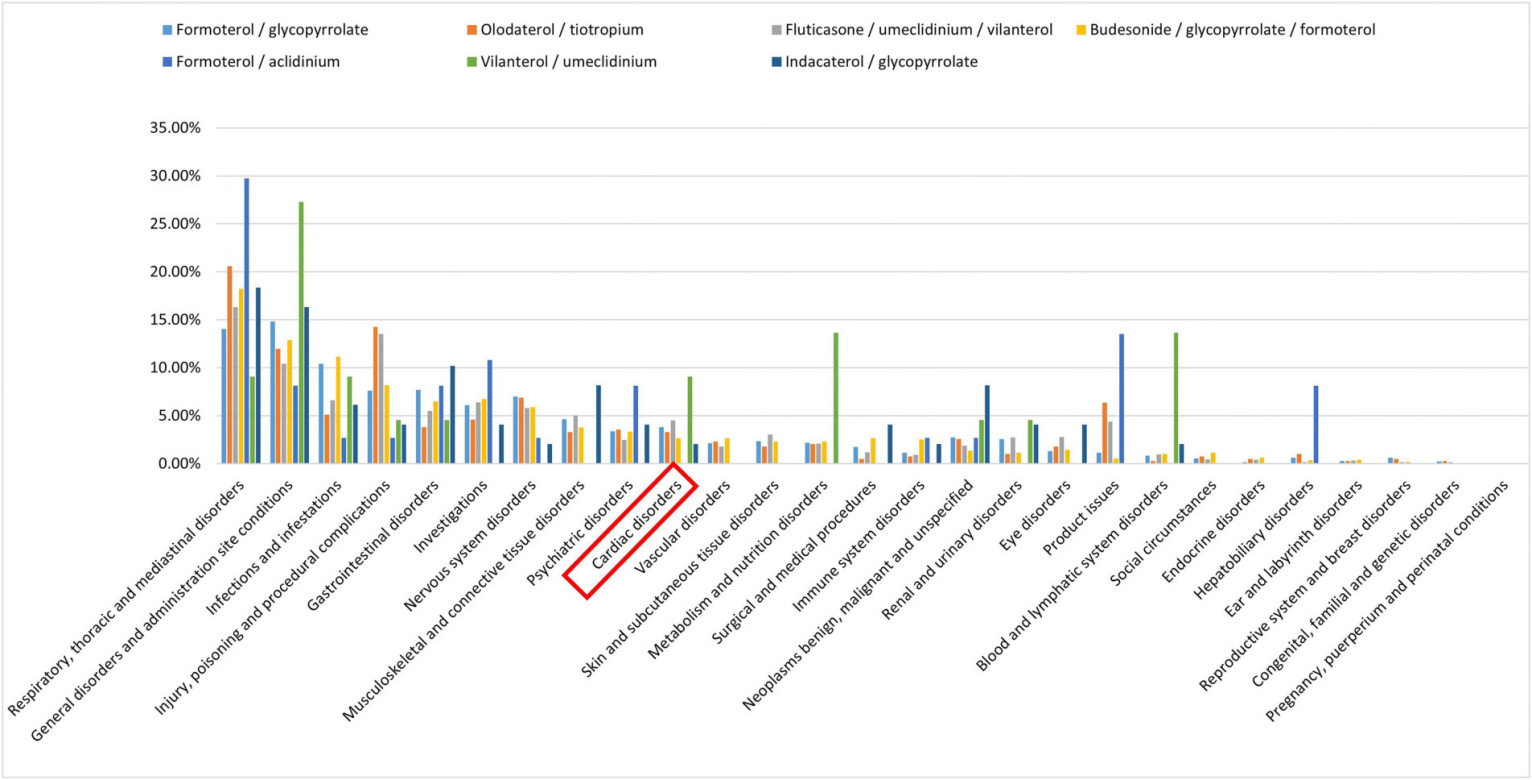
The percentage of ADEs classified by SOC level for each target drug compared to the total ADEs.

After processing the data, this study included 3,149 reports and 38,755 adverse events. Figure 1 illustrates the screening process for cardiac adverse events (CAEs) data related to the target drug in the FAERS database. Since the cardiotoxic adverse reactions of Formoterol/aclidinium, Indacaterol/glycopyrrolate, and Vilanterol/umeclidinium were minimal, they were excluded from the population characteristics analysis. In the final analysis, 3,120 reports on the use of ILABs were considered, which encompassed 653 adverse cardiac reactions (reported in 454 cases). The bar chart in Figure 1 presents the percentage of AEs classified by SOC level for each target drug relative to the total AEs. Across all target drugs, AEs signals spanned 27 SOCs, with CAEs emerging as the 10th most significant signal category. Notably, Vilantrol/umeclidinium had a markedly higher proportion of CAEs compared to other target drugs; however, given its small absolute number, these findings serve as a limited reference.

The characteristics of the population reporting cardiac toxicity caused by ILABs were shown in Table 2. The number of male and female cases was comparable; however, over 20% of the CAE cases in the FUV group had missing gender data, indicating a potential risk of reporting bias. Elderly patients constituted a substantial proportion of the study cohort, with a mean age of 70.5 years; however, missing age data were reported for approximately one-third of the cases. Notably, the mean age of CAEs onset in the BGF group was 10 years lower than the overall average. This discrepancy could not be ruled out as being associated with the small sample size of the BGF group and the 11.11% rate of missing age data in this subgroup. The report mainly came from North America and was primarily reported by consumers. Due to the possibility of multiple adverse reaction outcomes in a single report, we counted based on the most severe outcome. Death and hospitalization were the most common adverse reaction outcomes. FG had the lowest number of deaths, while FUV had the highest.

**Table 2:** Characteristics of reports with CAEs of compound ILABs sourced from the FAERS database.

Characteristics	Total (%)	Drug name			
		FG	OT	BGF	FUV
Total of cases, *n*	454	54	170	9	221
PT exceeding the signal threshold, *n*	551	74	158	3	294
Gender, *n* (%)					
Female	198 (43.61%)	33 (61.11%)	83 (48.82%)	7 (77.78%)	75 (33.94%)
Male	195 (42.95%)	19 (35.19%)	81 (47.65%)	2 (22.22%)	93 (42.08%)
Unkown	61 (13.44%)	2 (3.70%)	6 (3.53%)	–	53 (23.98%)
Age, years, *n* (%)					
19–65	101 (22.25%)	13 (24.07%)	43 (25.29%)	5 (55.56%)	40 (18.10%)
>65	209 (46.04%)	24 (44.45%)	103 (60.59%)	3 (33.33%)	79 (35.75%)
Unkown	144 (31.72%)	17 (31.48%)	24 (14.12%)	1 (11.11%)	102 (46.15%)
Mean	70.5	67.97	70.09	60.77	71.84
Reporting region, *n* (%)					
Europe	34 (7.49%)	2 (3.70%)	9 (5.29%)	–	23 (10.41%)
North America	406 (89.43%)	51 (94.44%)	155 (91.18%)	9 (100.00%)	191 (86.43%)
Asia	5 (1.10%)	–	1 (0.59%)	–	4 (1.81%)
South America	2 (0.44%)	–	–	–	2 (0.90%)
Oceania	6 (1.32%)	–	5 (2.94%)	–	1 (0.45%)
Unkown	1 (0.22%)	1 (1.85%)	–	–	–
Reporter's type of occupation, *n* (%)					
Consumer	226 (49.78%)	28 (51.85%)	84 (49.41%)	5 (55.56%)	109 (49.32%)
Health professional	78 (17.18%)	4 (7.41%)	21 (12.35%)	1 (11.11%)	52 (23.53%)
Physician	89 (19.60%)	11 (20.37%)	37 (21.76%)	1 (11.11%)	40 (18.10%)
Pharmacists	30 (6.61%)	2 (3.70%)	15 (8.82%)	–	13 (5.88%)
Others	16 (3.52%)	3 (5.56%)	11 (6.47%)	–	2 (0.90%)
Unkown	15 (3.30%)	6 (11.11%)	2 (1.18%)	2 (22.22%)	5 (2.26%)
Outcome of each PT, *n* (%)					
Total	656	88	213	13	342
Death	213 (32.47%)	6 (6.82%)	63 (29.58%)	2 (15.38%)	142 (41.52%)
Life-threatening	31 (4.73%)	3 (3.41%)	6 (2.82%)	–	22 (6.43%)
Hospitalization	288 (43.90%)	55 (62.50%)	116 (54.46%)	8 (61.54%)	109 (31.87%)
Disability	7 (1.07%)	1 (1.14%)	6 (2.82%)	–	–
Congenital anomaly	4 (0.61%)	–	–	–	4 (1.17%)
Other serious (Important Medical Event)	113(17.23%)	23(26.14%)	22(10.33%)	3(23.08%)	65(19.01%)

FG: Formoterol/glycopyrrolate, OT: Olodaterol/tiotropium, BGF: budesonide/glycopyrrolate/formoterol, FUV: Fluticasone/umeclidinium/vilanterol, PT: preferred term (in FAERS database).

### 3.2 Results of significant CAE signals

We tallied the various categories and frequencies of CAEs stemming from the inhalation of long-acting bronchodilators. Specifically, atrial fibrillation emerged as the most prevalent type (*N* = 79, accounting for 14.93% of cases), followed closely by myocardial infarction (*N* = 76, 14.37%), congestive heart failure (*N* = 58, 10.96%), cardiac disorder (*N* = 46, 8.70%), and cardiogenic shock (*N* = 43, 8.13%). Table 3 1presents the top 5 CAEs ranked by the number of occurrences in the total sample, the LABA/LAMA group, and the ICS/LABA/LAMA group.

**Table 3:** Top 5 cases of CAEs caused by compound ILABs.

Top	Total		LABA/LAMA		ICS/LABA/LAMA	
	PT	*n*	PT	*n*	PT	*n*
1	Atrial fibrillation	79	Atrial fibrillation	51	Cardiogenic shock	43
2	Myocardial infarction	76	Myocardial infarction	46	Arteriosclerosis coronary artery	40
3	Cardiac failure congestive	58	Cardiac failure congestive	33	Ventricular fibrillation	38
4	Cardiac disorder	46	Cardiac disorder	21	Myocardial infarction	30
5	Cardiogenic shock	43	Cardiac arrest	18	Atrial fibrillation	28

PT, preferred term (in FAERS database); ICS, inhaled corticosteroids; LABA, long-acting *β*-receptor agonist; LAMA, long-acting muscarinic antagonists.

**Figure 2:**
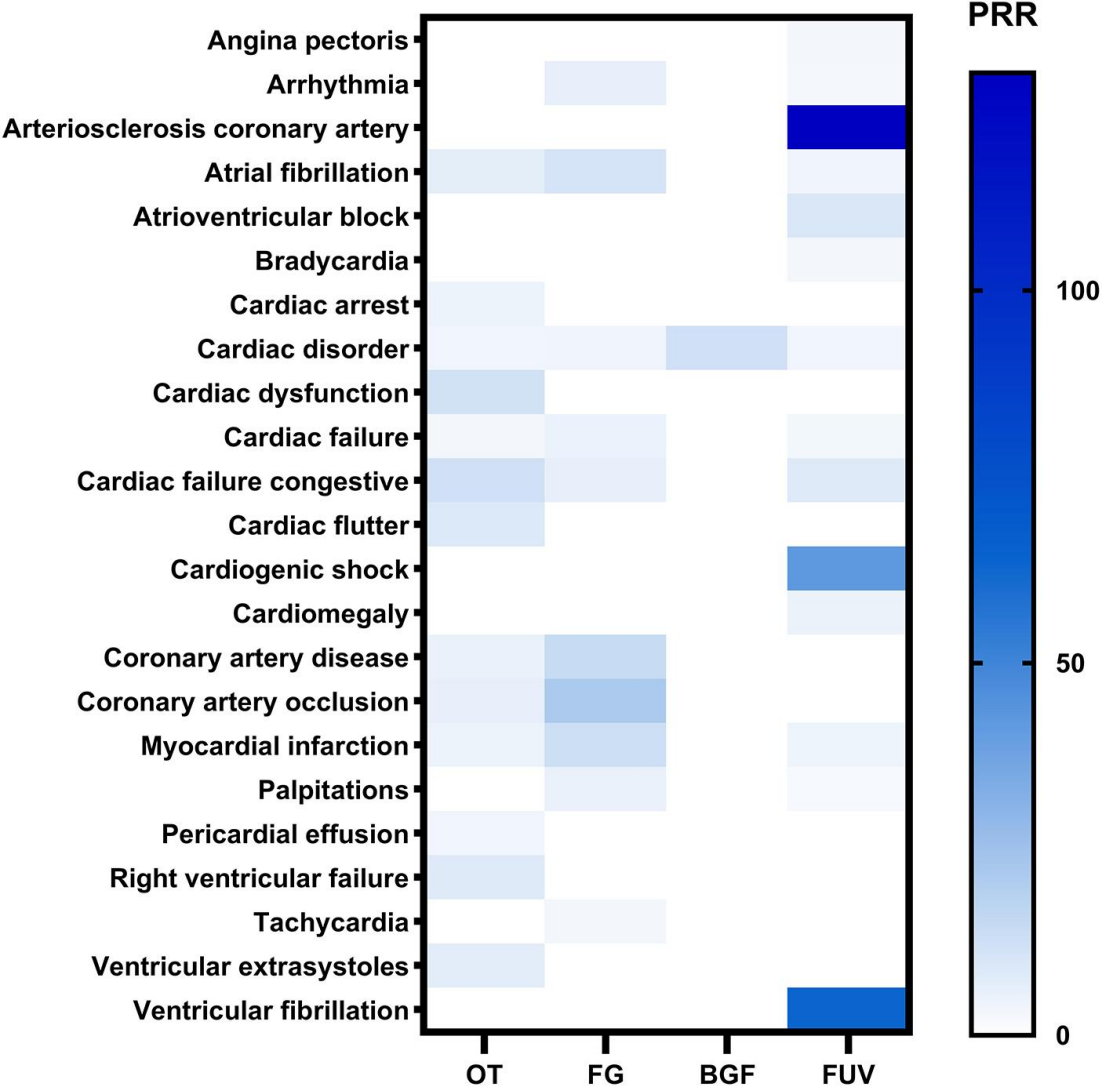
The heatmap shows the PRR for 23 CAEs (with cases no less than 3) in the FAERS database under different compound ILABs. FG, Formoterol/glycopyrrolate; OT, Olodaterol/tiotropium; BGF, budesonide/glycopyrrolate/formoterol; FUV, Fluticasone/umeclidinium/vilanterol; PRR, proportional reporting odds ratio.

**Figure 3:**
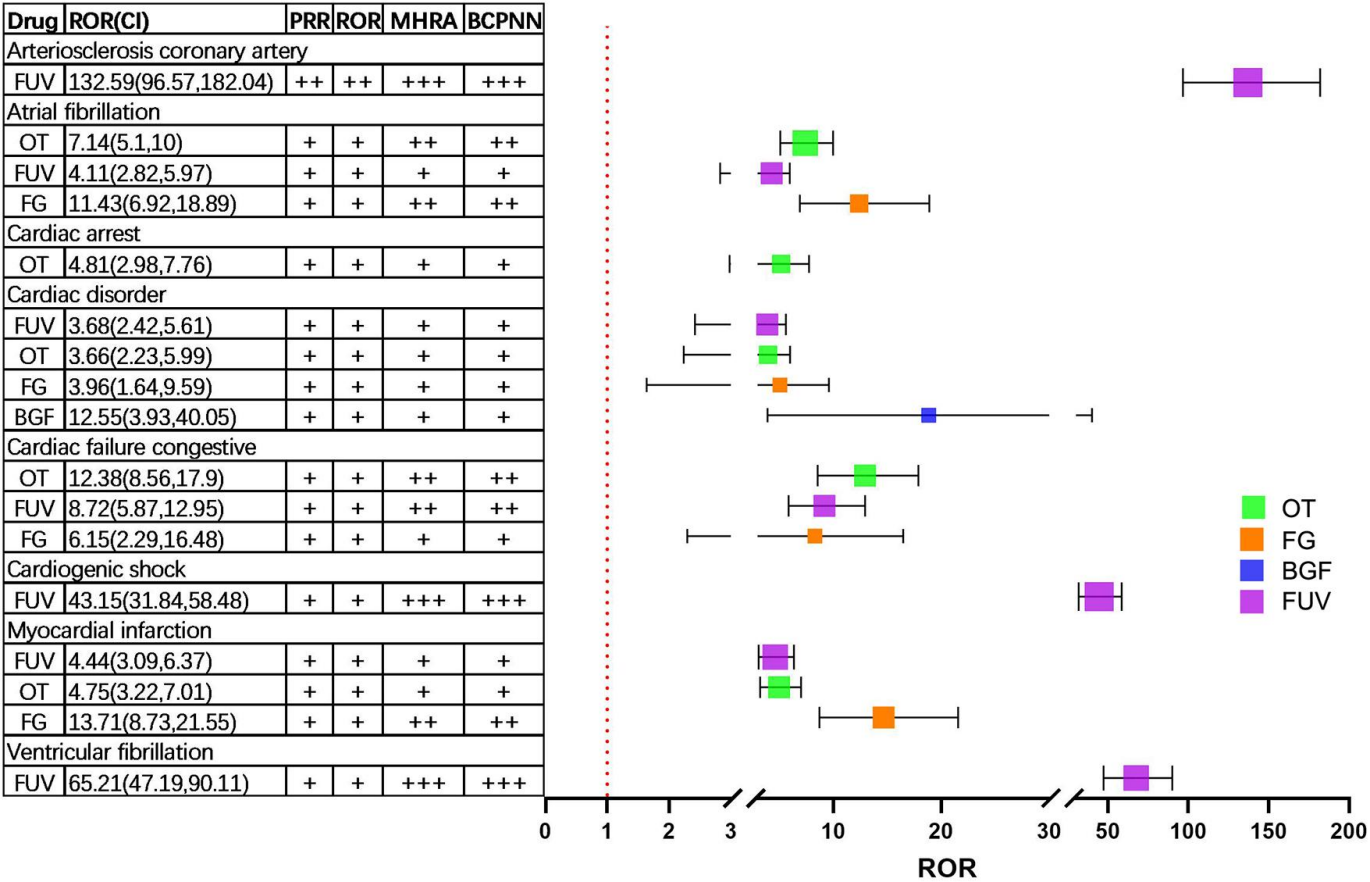
Signal intensity of CAEs under PRR, ROR, MHRA and BCPNN algorithms. FG, Formoterol/glycopyrrolate; OT, Olodaterol/tiotropium; BGF, budesonide/glycopyrrolate/formoterol; FUV, Fluticasone/umeclidinium/vilanterol; ROR, reporting odds ratio; PRR, proportional reporting ratio; MHRA, Medicines and Healthcare Products Regulatory Agency.

We conducted a disproportionality analysis using the entire FAERS database as a control, calculating the PRR of PT for a minimum of 3 cases in the CAE for each drug. Figure 2 illustrated the potential cardiac safety signal spectra across various drugs, utilizing heatmaps. The study encompassed 23 distinct categories of cardiac adverse events (PTs) with possible safety signals. In addition, PRR, ROR, MHRA and BCPNN algorithms were used to detect the signal intensity of CAEs in Figure 3. The results showed that the CAEs associated with FUV such as cardiogenic shock, arteriosclerosis coronary artery, and ventricular fibrillation were more significant signal. There was a significant difference in the risk of atrial fibrillation related to FG and FUV, and the former had a greater risk (ROR value: 11.43 vs. 4.11). The BCPNN algorithm was employed for signal detection of CAEs with a small number of occurrences (a < 3). All results were below the signal detection threshold, indicating the absence of clinically meaningful signals.

### 3.3 Subgroup analysis

We performed a subgroup analysis, examining various aspects such as composition, onset time, mortality outcomes, and adverse events.

#### 3.3.1 Subgroup analysis grouped by component types

Due to the low incidence of adverse events related to BGF in the FAERS database, the cardiac safety signals associated with the triple inhalation formulation in this study mainly came from FUV (which generated 14 potential cardiac safety signals). There were 16 categories of cardiac safety signals related to double bronchodilators. The top 5 CAEs related to triple inhalation formulations and double bronchodilators were shown in Table 3. Among them, the number of adverse events related to triple inhalation formulations, such as cardiogenic shock, arteriosclerosis coronary artery, and ventricular fibrillation, was significantly more than that of double bronchodilators, which had no relevant reports.

#### 3.3.2 Time to onset analysis

**Figure 4:**
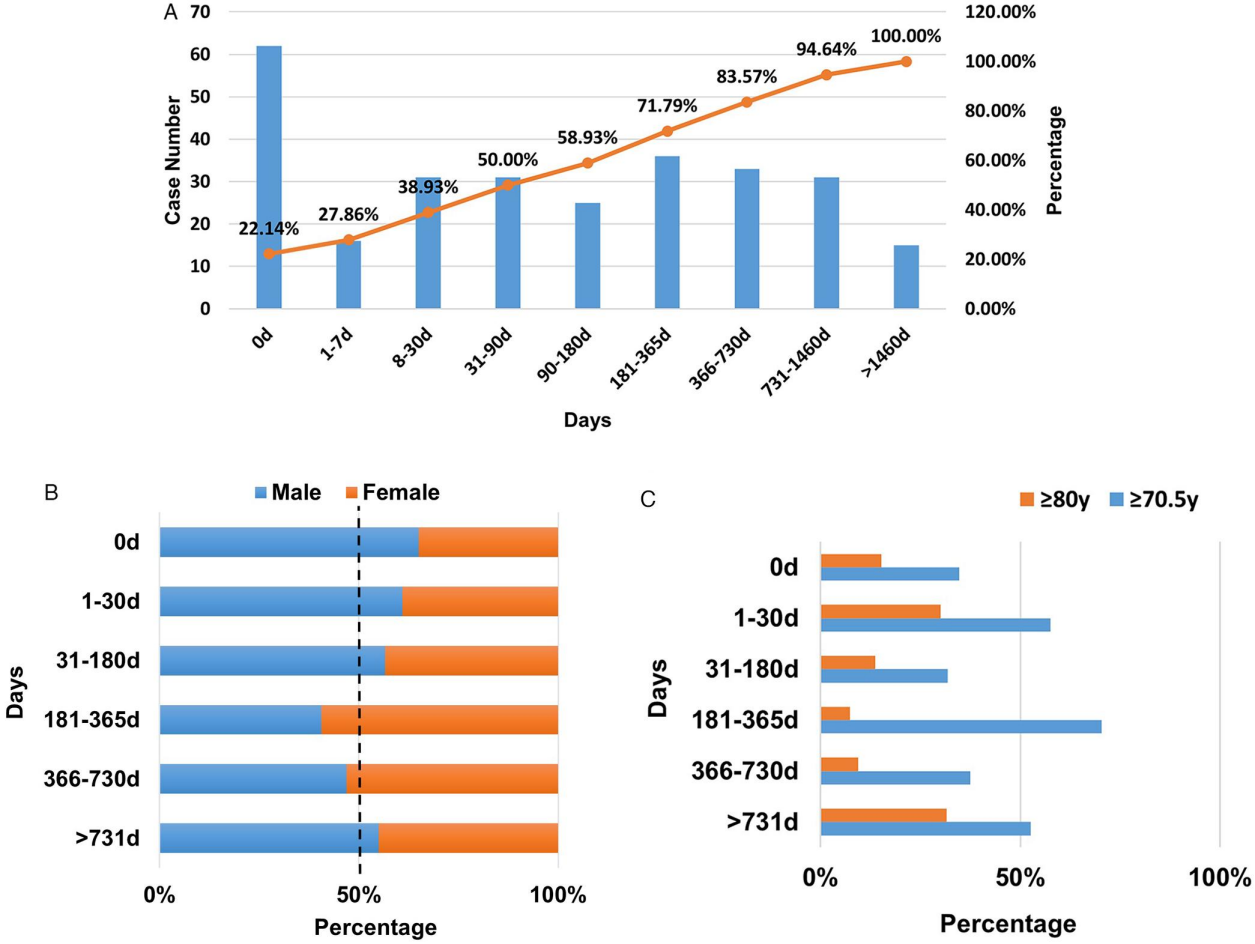
The time to onset of CAEs of compound ILABs. **(A)** Number of patients with CAEs at different onset time points and the cumulative curve of the proportion of patients. **(B)** Gender differences of patients across different onset time periods of CAEs. **(C)** Proportion of onset time periods of CAEs in patients aged over 70.5 years and over 80 years.

Due to incomplete data collection in some reports, only 280 cases of CAEs were included in the subgroup analysis of onset time, as shown in Figure 4A. The onset time was obtained from the start of medication to the occurrence of adverse events. Over 20% of CAEs surfaced on the day of drug administration (Day 0), with half manifesting within three months (90 days). Among the CAEs arising in the short term (within 90 days), Myocardial infarction, Cardiac failure, and Aerial fibrosis were the most prevalent. Specifically, on Day 0, the most common CAEs were Myocardial infarction, Cardiac failure, and Palpitations. For CAEs emerging over a prolonged period (exceeding 1 year), Myocardial infarction, Cardiac failure coalescence, and Cardiac disorder were predominant. Notably, both in the short and long term, myocardial infarction and heart failure stood out as significant ADE signals. Cardiomegaly was evident in long-term CAEs signals but absent in short-term signals. The most severe consequences of CAEs occurring within the first week were primarily hospitalization and other serious events. For CAEs occurring over 30 days, in addition to hospitalization and other serious events, death ranked third in frequency. Gender distribution remained consistent across different onset time periods, as illustrated in Figure 4B. Among patients over 70.5 years old (the average age in this study), the highest signal of cardiac toxicity was detected between 181 and 365 days (70.37%), followed by the period of 1–30 days (57.50%). For extremely elderly patients over 80, the highest cardiac toxicity signals were observed after 731 days (31.58%) and between 1 and 30 days (30.00%), as depicted in Figure 4C.

#### 3.3.3 Analysis of death outcome cases

**Figure 5:**
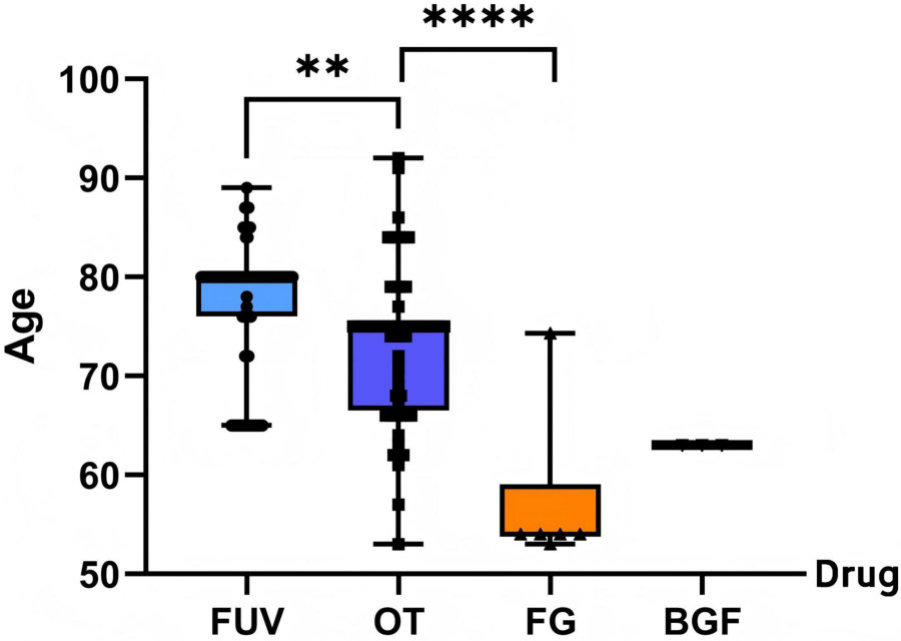
Age distribution of death cases related to compound ILABs, **P = 0.0011, ****P < 0.0001 of t-test, FG, Formoterol/glycopyrrolate; OT, Olodaterol/tiotropium; BGF, budesonide/glycopyrrolate/formoterol; FUV, Fluticasone/Umeclidinium/Vilanterol; ILABs, inhaled long-acting bronchodilators.

The drugs FUV and TO were reported to have the highest mortality outcomes among CAEs. In FUV related death reports, cardiogenic shock, ventricular fibrillation, and Arteriosclerosis coronary artery were identified as the strongest signaling cardiac toxicity events, while OT was identified as cardiac arrest Atrial fibrillation. The median (mean) ages of patients in death reports related to the overall cohort, OT, FG, BGF, and FUV groups were 74.76 (76.5), 75 (73.12), 54 (57.22), 63 (63), and 80 (77.11) years, respectively, as illustrated in Figure 5. The age of CAE-related death cases in the FG group was lower than that of the total sample, and the possibility of selective reporting in this group cannot be excluded. Data of the BGF group were derived primarily from one single death case, and thus the results lack statistical representativeness. Age data were missing for 33.10% of the fatal outcome cases in the FUV group; thus, the results presented in Figure 5 may be subject to potential bias.

#### 3.3.4 Other PTs co-reported with CAEs

**Figure 6:**
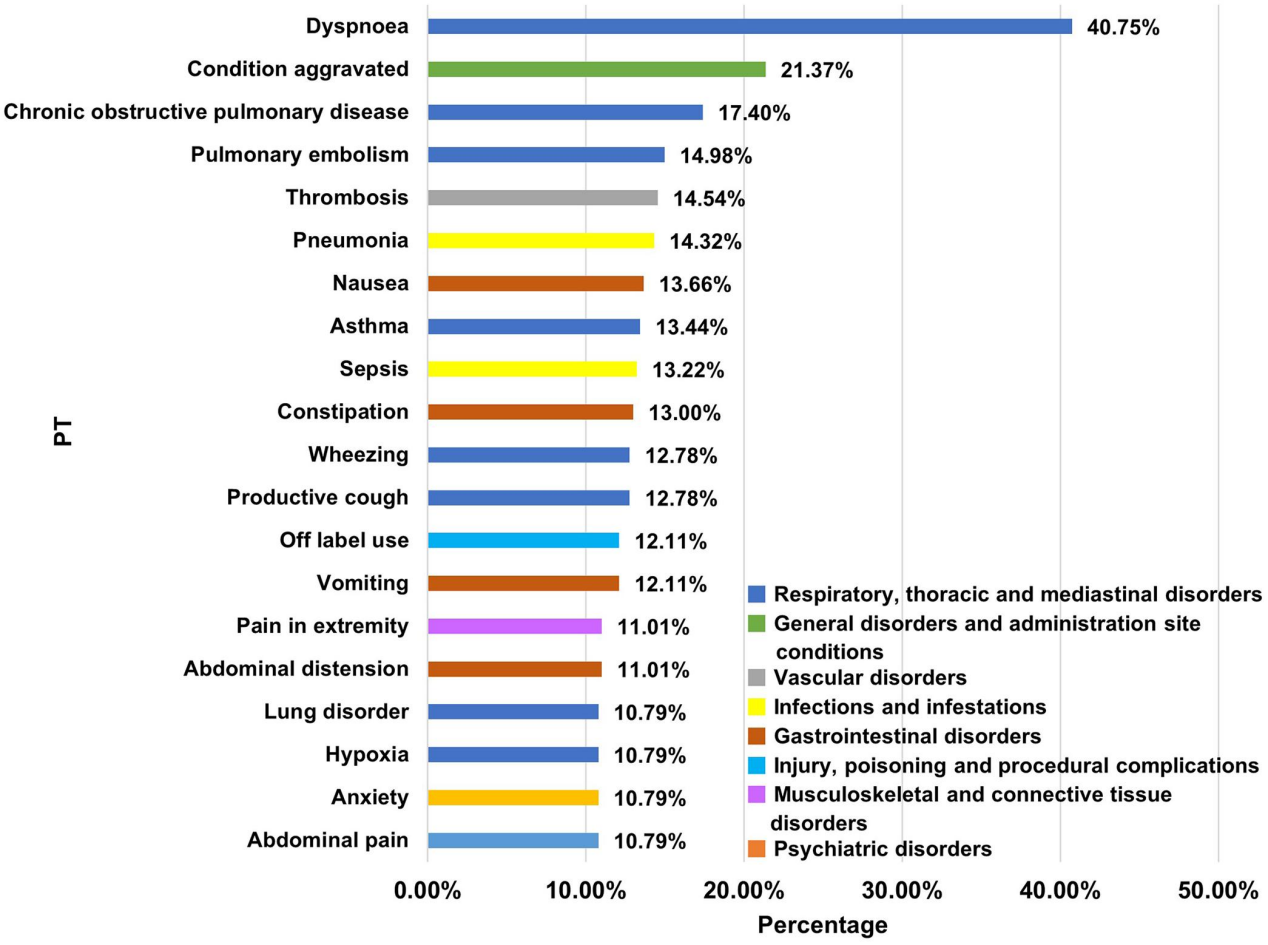
Commonly reported other ADEs of compound ILABs -related CAEs (ranked top 20).

An analysis was conducted on cases of cardiac toxicity accompanied by other types of ADE. 384 cases (84.58%) reported both CAE and other ADEs associated with the use of inhaled long-acting bronchodilators. Furthermore, in CAE reports resulting in death, 79.82% of the cases involved additional ADE categories. At the SOC level, a total of 25 distinct ADE categories were reported alongside cardiac disorders, with respiratory, thoracic and mediastinal disorders, general disorders and administration site conditions, investigations, infections and infestations, and nervous system disorders being the most prevalent five. From the PT level perspective, 864 types of PTs were identified as occurring concurrently with CAEs. Among these, dyspnoea, condition aggravated, chronic obstructive pulmonary disease, pulmonary embedding, and thrombosis ranked as the top five, as illustrated in Figure 6.

## 4 Discussion

This study verifies that compound ILABs have the potential to induce CAEs, including atrial fibrillation, myocardial infarction, congestive heart failure, cardiogenic shock, and various other cardiac events, echoing previous research findings (16). LABA primarily acts as a bronchodilator by activating *β*₂ receptors, while also stimulating cardiac *β*₁ receptors. This stimulation can elevate myocardial contractility, heart rate, and myocardial oxygen consumption, possibly triggering cardiac toxicity reactions like myocardial ischemia and arrhythmia. LAMA inhibits the inhibitory effect of the vagus nerve on the heart by blocking M-cholinergic receptors, leading to relative excitation of the sympathetic nervous system and potentially causing an increase in heart rate (17, 18). Therefore, from a pharmacological perspective, the cardiac side effects caused by LABA and LAMA have theoretical basis (19). Another study had shown that COPD patients who newly used LABA or LAMA had a 1.5-fold increase in cardiovascular risks such as arrhythmia, heart failure, and ischemic stroke within 30 days of treatment initiation, while new use of LABA/LAMA would increase by about 2-fold (4).

Our study did not include a comparison between compound ILABs and LAMA monotherapy primarily due to its reliance on relevant studies conducted by Matera et al. (20). This study also utilized the FAERS database to investigate the occurrence of CAEs in patients using LAMA. The results showed that the risk of CAEs was higher with LAMA alone than glycopyrronium/indacaterol, glycopyrronium/formoterol, umeclidinium/vilanterol and triple preparations. But different clinical studies had shown different research results. For instance, Lee et al. observed that the occurrence of tachyarrhythmias was comparable when using LABA or LAMA as monotherapy (21). Dong et al. reported that the CAEs were analogous to those observed with the combined LABA/LAMA regimen (22). The study by Rebordosa et al. showed that the risk of acute coronary syndrome, stroke, and major adverse cardiac events among current users of aclidinium, aclidinium/formoterol, tiotropium bromide, other LAMA, LAMA/LABA, or LABA/ICS was similar to that of current users of LABA (23). Unlike the above results, Parkin et al.'s study showed that LABA/LAMA significantly increased the risk of acute coronary syndrome compared to LAMA (24, 25), while Suissa et al.'s results showed that the combination of ILABs increased the risk of heart failure compared to monotherapy (26). Although there were differences in the risk comparison of CAEs between single and compound ILABs in the above studies, they all confirmed the risk of ILABs causing CAEs.

COPD exhibits a strong association with cardiovascular disease, as evidenced by the fact that roughly 64% of COPD patients also grapple with cardiovascular conditions. Moreover, approximately one-third of COPD patients succumb to cardiovascular disease (27). A previous study indicated that COPD patients face an elevated risk of CAEs, a risk that was tied to their cardiovascular disease history and frequent exacerbations, rather than their inhalation of bronchodilators (28). Due to the limited information in the farse database and the limitations of this study, especially the inability to obtain the diagnosis and co-medication of patients when they first took drugs, we did not conduct a subgroup analysis of COPD patients with cardiovascular disease. Therefore, it was impossible to identify and exclude the confounding factors of cardiovascular problems in underlying diseases. In the future, real-world research or systematic reviews will be further carried out.

This study performed a subgroup analysis to assess the time-dependent risk of CAEs, revealing that more than half occurred within the first 3 months. Notably, patients aged over 80 years exhibited the greatest risk during the initial 1–30 days of medication and after 731 days. These findings imply acute reactions and potential long-term cardiac toxicity; however, the elevated long-term risk does not preclude the influence of aging or disease progression. The number of CAEs on the day of administration (day 0) accounted for more than 20% of all CAEs, which did not exclude that it was related to the report filling method or report bias. Furthermore, a nested case-control study examined the heart disease risk in COPD patients using LABA/LAMA compared to those who did not. The results showed that the risk of CAEs in patients who used LABA/LAMA within 30 days was significantly higher than that in patients who did not use it (OR 1.16, 95% CI 1.05–1.28, *P* = 0.003), but there was no significant difference among patients who used LABA/LAMA within 30–90 days, 90–180 days, and>180 days (4). This result suggested that caution should be exercised when using LABA/LAMA for short-term cardiac toxicity risks, which was consistent with the findings of this study. However, there were inconsistencies between the results of this study and some clinical trials. A study evaluated electrocardiograms in elderly COPD patients before, 15 min, and 60 min of use of IG, and no significant abnormal electrocardiogram were observed. However, the time limit for this study was too short, making it difficult to identify the risks of CAEs and chronic heart disease caused by repeated medication (29). A case-control study found that COPD patients who used LAMA/LABA or triple inhalation formulations for ≥90 days, ≥180 days, and ≥360 days had no significant increase in the risk of CAEs (28). Yet, the study did not investigate the risk of CAEs occurring within 30 days of medication use. Hence, further real-world research is warranted to comprehensively investigate the risk of CAEs across different age groups, medication durations, and other factors.

This study found that the proportion of CAE reports for triple therapy and double bronchodilators is similar, but the number of events related to triple therapy such as cardiogenic shock, arteriosclerosis coronary artery, and ventricular fibrillation was higher. Numerous clinical studies had contrasted ICS/LABA treatment regimens, revealing that triple therapy was independently linked to the onset of ischemic heart disease, heart failure, arrhythmia, and atrial fibrillation/spread (30), whereas LAMA/LABA therapy exhibited a similar incidence of CAEs to ICS/LABA (28, 31). Therefore, the indirect comparison results indicated that the triple therapy had a higher risk of CAEs compared to the double bronchodilator regimen. Furthermore, the phase III, 52-week ETHOS trial compared the risk of first CAEs between BGF and FG, demonstrating a higher risk for the latter (32). Therefore, COPD patients using triple preparations should be more alert to the risk of cardiotoxicity, but the specific types of cardiotoxicities caused by triple preparations are different in different studies, and further research is needed.

This study had certain limitations. Firstly, the data came from a spontaneous reporting system, which might have issues of underreporting and reporting bias, leading to discrepancies between research results and actual clinical situations. The gender and age data of patients with CAEs were missing to varying degrees, which had uncertain impact on the reliability of the results. At present, no evidence had been obtained that sex and age were independent factors of CAEs caused by inhaled agents. At the same time, due to the large amount of missing data about the onset time of CAEs in the original report, the research results of “time to onset analysis” may be at risk of publication bias. Secondly, COPD patients often have complications such as cardiovascular disease, and elderly patients often have multiple drug use (33). This study did not exclude these confounding factors, which might affect the accuracy of the research results. To comprehensively evaluate the cardiovascular safety of ILABs, it was recommended to carry out the following work in the future: (1) conduct a large sample prospective cohort study to systematically compare the differences in cardiovascular risk among different ILAB regimens; (2) Establish a risk prediction model based on multidimensional clinical features to provide personalized treatment decision support for COPD patients with cardiovascular disease. These studies will help to more accurately evaluate the clinical benefit risk ratio of ILABs and optimize treatment options.

## 5 Conclusion

This study conducted pharmacovigilance analysis on CAEs related to compound ILABs using the FAERS database. The results confirmed the risk signals of CAEs, such as causing atrial fibrillation, myocardial infarction, heart failure, etc., especially in the early stages of medication. The overall CAEs risk signals of triple inhalation formulations (mainly FUV) were comparable to that of double bronchodilators, but more attention should be paid to the occurrence of events such as cardiogenic shock, arteriosclerosis coronary artery, and ventricular fibrillation. Clinical recommendations include regular monitoring of electrocardiogram and cardiac function for COPD patients, especially the elderly and those with underlying cardiovascular diseases. The elderly population should pay more attention to the cardiac toxicity of short-term/long-term medication.

## Data Availability

Publicly available datasets were analyzed in this study. This data can be found here: https://fis.fda.gov/extensions/FPD-QDE-FAERS/FPD-QDE-FAERS.html.
