# Supplementary material for: Cardiac disorders associated with compound long-acting bronchodilators for inhalation: a pharmacovigilance analysis of the FDA adverse event reporting system database
Source: Front Cardiovasc Med. 2026 Mar 9;13:1715192. doi: 10.3389/fcvm.2026.1715192 (PMC13006220; doi:10.3389/fcvm.2026.1715192)
Supplement: Supplementary file 1 [file Table1.docx]

Supplementary Material

# Supplementary Tables 1

Fourflod table of disproportionality analysis

| **Drug** | **Number of the target ADEs** | **Number of the non-target ADEs** | **Total** |
| --- | --- | --- | --- |
| Target drug | a | b | a+b |
| Non-target drugs | c | d | c+d |
| Total | a+c | b+d | N= a+b+c+d |

Abbreviation: ADEs, adverse drug events; a, the count of target ADEs linked to the target drug; b, non-target ADEs for the same drug; c, target ADEs attributed to non-target drugs in the FAERS database; d, non-target ADEs caused by non-target drugs.

The calculation formula of each index is as follows:

# 1.1 Analysis algorithms of PRR

PRR=$\frac{a/(a+b)}{c/(c+d)}$ (1)

SE(lnPRR)= $\sqrt{(\frac{1}{a}-\frac{1}{a+b}+\frac{1}{c}-\frac{1}{c+d})}$ (2)

95%CI（PRR）=$e^{ln(PRR)}$^±1.96^$\sqrt{(\frac{1}{a}-\frac{1}{a+b}+\frac{1}{c}-\frac{1}{c+d})}$ (3)

# 1.2 Analysis algorithms of ROR

ROR=$\frac{(a/c)}{(b/d)}=\frac{\mathrm{ad}}{\mathrm{bc}}$ (4)

SE(lnROR)= $\sqrt{(\frac{1}{a}+\frac{1}{b}+\frac{1}{c}+\frac{1}{d})}$ (5)

95%CI(ROR)=$e^{ln(ROR)}$^±1.96^$\sqrt{(\frac{1}{a}+\frac{1}{b}+\frac{1}{c}+\frac{1}{d})}$ (6)

# 1.3 Analysis algorithms of MHRA

PRR=$\frac{a/(a+b)}{c/(c+d)}$ (7)

χ^2^=$\frac{({\mid\mathrm{ad}-\mathrm{bc}\mid(a+b+c+d)/2)}^{2} (a+b+c+d)}{(a+b)(a+c)(c+d)(b+d)}$ (8)

# 1.4 Analysis algorithms of BCPNN

IC=log_2_ $\frac{a(a+b+c+d)}{(a+b)(a+c)}$ (7)

E(IC)= log_2_ $\frac{(a+\gamma11)(a+b+c+d+\alpha)(a+b+c+d+\beta)}{(a+b+c+d+\gamma)(a+b+\alpha1)(a+c+\beta1)}$ (8)

V(IC)=$\frac{1}{{(ln2)}^{2}}($ $\frac{a+b+c+d-a+\gamma-\gamma11}{(1+a+b+c+d+\gamma)(a+\gamma11)}$ _+_ $\frac{a+b+c+d-a-b+\alpha-\alpha1}{(a+b+\alpha1)(1+a+b+c+d+\alpha)}$ _+_ $\frac{a+b+c+d-a-c+\beta-\beta1}{(a+c+\beta1)(1+a+b+c+d+\beta)}$ ) (9)

γ=γ11$\frac{(a+b+c+d+\alpha)(a+b+c+d+\beta)}{(a+b+\alpha1)(a+c+\beta1)}$ (10)

IC-2SD=E(IC)-2$\sqrt{V(IC)}$ (11)

α1=β1=1, α=β=2, γ11=1

# 1.5 Detection threshold of signal and judgment standard of signal strength grade

| Signal detection method | Signal threshold | Signal Strength | | |
| --- | --- | --- | --- | --- |
|  |  | + | ++ | +++ |
| PRR | a≥3且PRR-1.96SE(lnPRR)＞1 | 1＜PRR-1.96SE(lnPRR)＜50 | 50≤PRR-1.96SE(lnPRR) <1000 | 1000≤PRR-1.96SE(lnPRR) |
| ROR | a≥3且ROR-1.96SE(lnROR)＞1 | 1＜ROR-1.96SE(ln ROR)＜50 | 50≤ROR -1.96SE(ln ROR ) <1000 | 1000≤ROR -1.96SE(ln ROR) |
| MHRA | a≥3、PRR≥2且χ2≥4 | 4≤χ^2^<100 | 100≤χ^2^<1000 | 1000≤χ^2^ |
| BCPNN | (IC-2SD) > 0 | 0<(IC-2SD)<1.5 | 1.5≤(IC-2SD)<3 | 3≤(IC-2SD) |
